# The Sertoli cell: what can we learn from different vertebrate models?

**DOI:** 10.21451/1984-3143-AR2018-125

**Published:** 2020-05-22

**Authors:** Nathália de Lima e Martins Lara, Guilherme Mattos Jardim Costa, André Felipe Almeida Figueiredo, Luiz Renato de França

**Affiliations:** Laboratory of Cellular Biology, Department of Morphology, Federal University of Minas Gerais, Belo Horizonte, MG, Brazil.

**Keywords:** Sertoli cell, vertebrates, amniotes, anamniotes, spermatogenesis

## Abstract

Besides having medical applications, comparative studies on reproductive biology are very useful, providing, for instance, essential knowledge for basic, conservation and biotechnological research. In order to maintain the reproductive potential and the survival of all vertebrate species, both sperm and steroid production need to occur inside the testis. From the approximately fifty thousand vertebrate species still alive, very few species are already investigated; however, our knowledge regarding Sertoli cell biology is quite good. In this regard, it is already known that since testis differentiation the Sertoli cells are the somatic cells in charge of supporting and orchestrating germ cells during development and full spermatogenesis in adult animals. In the present review, we highlight key aspects related to Sertoli cell biology in vertebrates and show that this key testis somatic cell presents huge and intrinsic plasticity, particularly when cystic (fish and amphibians) and non-cystic (reptiles, birds and mammals) spermatogenesis is compared. In particular, we briefly discuss the main aspects related to Sertoli cells functions, interactions with germ cells, Sertoli cells proliferation and efficiency, as well as those regarding spermatogonial stem cell niche regulation, which are crucial aspects responsible for the magnitude of sperm production. Most importantly, we show that we could greatly benefit from investigations using different vertebrate experimental models, mainly now that there is a big concern regarding the decline in human sperm counts caused by a multitude of factors.

## Introduction

Since testis differentiation, in all vertebrate species so far investigated the Sertoli cells are the somatic cells in charge of supporting and orchestrating germ cells during their development and full spermatogenesis in adult animals (Pudney, 1993; Hess and França, 2005; Oatley *et al*., 2011; Griswold, 2015; França *et al*., 2016). Therefore, the total number of Sertoli cells per testis, as well as their proper interactions with germ cells and the number of these cells per Sertoli cell (Sertoli cell efficiency), are the key qualitative and quantitative determinants of sperm production (Sharpe, 1994; Hess and França, 2007; Griswold, 2015; França *et al*., 2016). In the present review, we concisely discuss and compare the key aspects related to Sertoli cell biology in different vertebrate groups, such as anamniotes (fish and amphibians) and amniotes (reptiles, birds and mammals), where respectively cystic and non-cystic spermatogenesis are observed (França *et al*., 2015, 2016).

## Sertoli cell discovery and morphology

The testis is the male gonad, where steroidogenesis and sperm production take place. Spermatogenesis is a highly organized process in which the germ cells go through several divisions and intricate differentiation steps, resulting in the production of the spermatozoa. This process is orchestrated mainly by the Sertoli cell that were named after Enrico Sertoli, author of the first publication reporting their existence (Sertoli, 1865; Ebner, 1888; Hess and França, 2007). In this publication, Enrico Sertoli describes their morphology “not unlike trees”, including details such as their bifurcated branches of cytoplasm, the niches where germ cells fit and the large nucleolus, apart from calling them “mother cells”, anticipating the comprehension of their crucial functions (Sertoli, 1865; Hess and Vogl, 2015).

**Figure 1:**
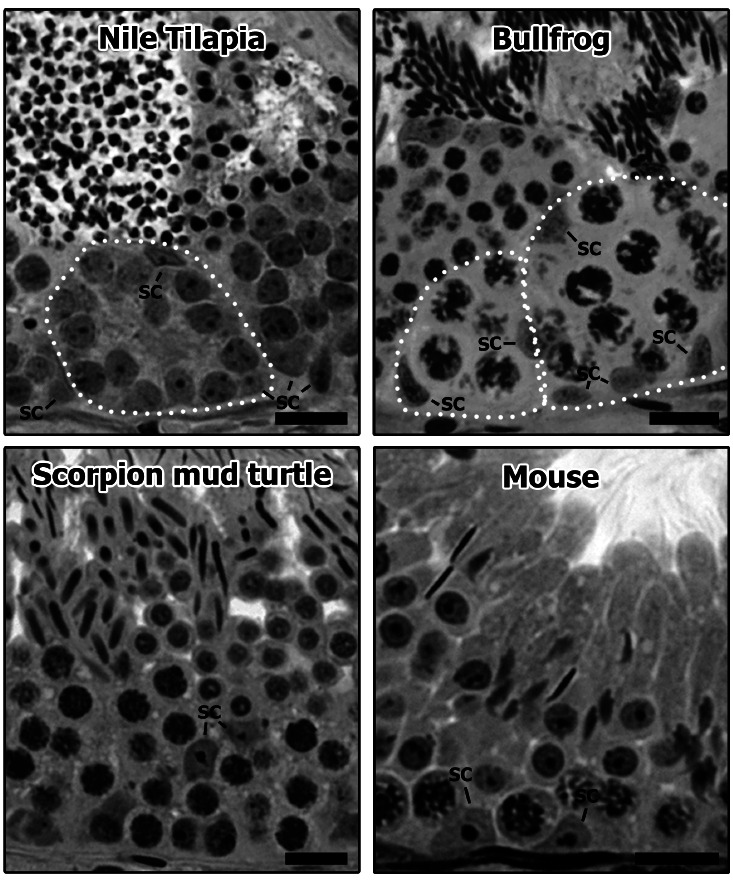
Histological sections from the seminiferous epithelium in different vertebrate species presenting cystic [Nile Tilapia (*Oreochromis niloticus*) and bullfrog (*Lithobates catesbeianus*)] or non-cystic [Scorpion mud turtle (*Kinosternon scorpioides*) and mouse (*Mus musculus*)] spermatogenesis arrangements. As it can be observed, the germ cell clones in the cystic arrangement are completely enveloped by the Sertoli cells (SC; white dotted lines), whereas in non-cystic arrangement one single Sertoli cell contacts several different germ cell types, despite exhibiting conspicuous structural polarity. Bars = 15 µm.

Using advanced staining techniques and electron microscopy, many decades later the details of the Sertoli cells morphology and function were shown and the understanding of this complex cell’s role in the testis morphophysiology have been a major topic in male reproduction research. Therefore, numerous reviews (Regaud, 1899; Elftman, 1963; Fawcett, 1975; Clermont *et al*., 1987; Sharpe, 1988; de Kretser, 1990; Clermont, 1993; Russell, 1993a,b; Vogl *et al*., 1993; Kerr, 1995; Griswold, 1998; Walker, 2003a,b; Wong and Cheng, 2009; Vogl *et al*., 2013; Ramaiah and Wilkinson, 2015; Griswold, 2016; França *et al*., 2016) and books (Russell and Griswold, 1993; Skinner and Griswold, 2005; Griswold, 2015) have been published describing the Sertoli cell morphology and functions, mostly focusing on mammals. However, due to the cystic versus non-cystic arrangement of spermatogenesis, distinct Sertoli cell characteristics are observed when comparing different vertebrate species [anamniotes (fish and amphibians) and amniotes (reptiles, birds and mammals)] (Please see Section 4 and Figure 1) (Russell and Griswold, 1993; Griswold, 1998; Hess and França, 2007; Schulz *et al*., 2010; França *et al*., 2015, 2016). 

Reflecting their relationship with germ cells, in overall the Sertoli cell shape may vary according to the species and the progression of spermatogenesis. Considerable variations are also observed for the expression of proteins and growth factors, which also change according to the age of development and seasonality (Russell, 1993a,b; Rothbarth *et al*., 2001; França and Chiarini-Garcia, 2005; Hess and Vogl, 2015; França *et al*., 2016). Additionally, as the germ cell requirements, interactions and metabolic needs change substantially, high variations are observed on the Sertoli cell cytoplasm extension, the amount of nuclear pores, the presence and translocation of organelles and the protein expression pattern and location across the different phases of spermatogenesis (Toppari *et al*., 1991; França *et al*., 1993; Cavicchia *et al*., 1998; Johnston *et al*., 2008; Wright, 2015). To illustrate the above-mentioned variation on Sertoli cells characteristics, due to their endocytic activity in the elimination of residual bodies, an increase in the amount of lipid and lysosomes is usually observed in the Sertoli cells cytoplasm after spermiation (Ye *et al*., 1993; França *et al*., 1995; Hess and França, 2005). Structural characteristics of the Sertoli cells also varies among species, such as the heavily vacuolated nucleolus present in some ruminants (Pawar and Wrobel, 1991; Steger and Wrobel, 1994), the nucleus localization in the middle of the seminiferous epithelium in monkeys (Hess and França, 2005), the presence of Charcot-Bottcher cristaloids in men (Czaker, 1994; França and Chiarini-Garcia, 2005), and the presence and amount of lipid droplets and glycogen in the Sertoli cell cytoplasm (Fouquet, 1968; Russell, 1993a,b; Tedde *et al*., 1993; Erkan and Sousa, 2002).

## Proliferation and maturation

It is widely accepted that the number of Sertoli cells per testis and the Sertoli cell efficiency (which is measured as the number of germ cells per Sertoli cell) are the main determinants of the sperm production of a given species (Sharpe, 1994; Hess and França, 2007; Griswold, 2015; França *et al*., 2016). In this regard, the Sertoli cell proliferation that usually ends before puberty is a crucial event for testis physiology (Lara *et al*., 2018b). In laboratory rodents (such as rats and mice), this proliferation occurs mainly during fetal life, reaching its maximum activity just before birth (Orth, 1982, 1993; McCoard *et al*., 2003; França *et al*., 2016). However, the postnatal Sertoli cell mitotic activity is highly variable according to the species, lasting for instance 2-3 weeks in laboratory rodents. In humans, this somatic cell proliferate during the perinatal and neonatal period, becoming quiescent for several years and having a second peak of proliferation just before puberty (Cortes *et al*., 1987; Sharpe *et al*., 2003; Tarulli *et al*., 2012). In a scale of months or few years, a similar pattern is also observed in pigs, primates and cattle (Gondos and Berndston, 1993; França *et al*., 2000; Cooke *et al*., 2005).

In most studied species, FSH and androgens are considered important factors that regulate Sertoli cell proliferation (Skinner and Griswold, 2005; Lara *et al*., 2018b). Other factors usually associated with this process are estrogens, activins, TGF-beta, BMPs, interleukins and TNFalpha (Puglisi *et al*., 2004; Tarulli *et al*., 2012; Lucas *et al*., 2014a,b). After the fetal and postnatal periods of mitotic divisions, which are rather variable among different species, Sertoli cells stop proliferating and start to differentiate around puberty, being therefore able to support full spermatogenesis. This maturation process correlates for instance with the initiation of meiosis, the establishment of the Sertoli cell barrier and fluid secretion/lumen formation, which are clear signs of Sertoli cells maturation (Griswold, 2015; França *et al*., 2016). 

Based mainly in studies developed in laboratory rodents (rats and mice), thyroid hormones are considered a key factor in the regulation of Sertoli cell differentiation/maturation (Cooke *et al*., 2005). In these studies, it has been shown that neonatally induced hypothyroidism prolong the proliferative period and delay Sertoli cell maturation, increasing therefore their final number and, consequently, the testis size and the magnitude of sperm production (Van Haaster *et al*., 1992; Joyce *et al*. 1993; Auharek and França 2010; Lara and França, 2017). In an opposite way, hyperthyroidism accelerates Sertoli cell maturation, resulting in a smaller population of this cell in the testis, as well as smaller testis size and lower sperm production (Van Haaster *et al*., 1993; Cooke *et al*., 2005; Auharek and França, 2010). Similar results regarding thyroid hormones effects on testis size and sperm production were observed in chicken and the Nile tilapia (Kirby *et al*., 1996; Matta *et al*., 2002). However, paradoxically, in pigs thyroid hormones seem to regulate Sertoli cells in an opposite way. In this regard, postnatally induced hypothyroidism significantly decreased the number of Sertoli cells, testis size and sperm production, whereas a higher dose of thyroid hormone (T3) augmented the number of Sertoli cells per testis (Tarn *et al*., 1998; Silva-Jr, 2000; Klobucar *et al*., 2003). In bulls, no effects on Sertoli cells number were observed after the induction of neonatal hypothyroidism, whereas human studies suggested that decreased thyroid hormones might be associated to testicular enlargement (Jannini *et al*., 1995; Cooke *et al*., 2005). There is still no explanation regarding the observed inconsistencies of thyroid hormones effects among the different species of amniotes (reptiles, birds and mammals). One of the possibilities might be related to the aforementioned differences on Sertoli cell proliferation pattern. The expression of thyroid hormone receptors and/or interactions with other testis somatic cell types (i.e Leydig and peritubular myoid cells) should also not be excluded.

In relation to anamniotes, mainly due to continuous body growth, it has been shown that the Sertoli cell population is more dynamic, and that these cells remain mitotically active even after sexual maturity (Bouma and Cloud, 2005). Particularly, during spermatogenesis progression the number of Sertoli cells enveloping an individual spermatogenic cyst increases along with the germ cell number within that cyst. Interestingly, coincident with the formation of tight junctions between Sertoli cells, and similar to the establishment of puberty in mammals, usually the Sertoli cell number per cyst stabilizes after meiosis is complete and spermiogenesis initiates (Matta *et al*., 2002; Vilela *et al*., 2003; Schulz *et al*., 2005; Leal *et al*., 2009; França *et al*., 2016). As it occurs in mammals, FSH seems to be the main factor involved in Sertoli cell proliferation in anamniotes (Schulz *et al*., 2010, 2012; França *et al*., 2016). Also, as hypothyroidism increase Sertoli cell number in tilapia (Matta *et al*., 2002), thyroid hormones may also be important for Sertoli cell proliferation in fish (Matta *et al*., 2002; Morais *et al*., 2013). However, in contrast to mammals, thyroid hormones stimulate Sertoli cell proliferation in zebrafish (Morais *et al*., 2013). Recently, França *et al*. (2015) proposed two modes of Sertoli cell proliferation in fish. In the first, Sertoli cell proliferate to provide new niches for spermatogonial stem cells and to form new spermatogenic cysts (Morais *et al*., 2013; França *et al*., 2015). In the second mode, Sertoli cells already enveloping an existing cyst would divide, in order to accommodate the increase in the germ cell number until meiosis is complete and Sertoli cell maturation occurs (Billard and Breton, 1978; Almeida *et al*., 2008; França *et al*., 2015). An interesting aspect that could contribute to advance our understanding on Sertoli cell proliferation/maturation in fish is the observation that one Sertoli cell may be in contact with different cysts of germ cells in different phases of spermatogenesis (França *et al*., 2016).

In mammals, indicating the final maturation of this cell type and its capability to support full spermatogenesis is the establishment of Sertoli cell barrier, a remarkable feature that occurs around puberty (Cheng and Mruk, 2012; Griswold, 2015; Lara *et al*., 2018a). This barrier, observed close to the basement membrane, is formed by specialized junctional complexes between adjacent Sertoli cells and helps to protect the germ cells undergoing meiosis from an autoimmune reaction; creating therefore an immune privileged environment within the seminiferous tubules (Tung and Fritz, 1978; Francavilla *et al*., 2007; França *et al*., 2012; França *et al*., 2016). The main component of this barrier are tight junctions that divide the seminiferous epithelium in two compartments: basal and adluminal, where respectively early (spermatogonia and young spermatocytes) and late (more advanced spermatocytes and spermatids) germ cells are located. Due to the complex cyclic dynamics of the Sertoli cells barrier and the formation of a transient intermediate compartment, young primary spermatocytes move across these compartments without causing any damage to the junctional structure and testis physiology (Cheng and Mruk, 2012; França *et al*., 2012). Other components of this important barrier include gap junctions, desmosomes, and two types of adherens junctions that are testis-specific (tubulobulbar complexes and ectoplasmic specialization) (Lee and Cheng, 2004; Vogl *et al*., 2008; Cheng *et al*., 2011; Vogl *et al*., 2013; Lara *et al*., 2018a). Overall, these structural components make the Sertoli cell barrier one of the tightest in mammals (Mital *et al*., 2011; Cheng and Mruk, 2012). 

Regarding other vertebrate groups, there are relatively few studies related to the Sertoli cell barrier, and it is already known that the junctional complexes constituents are highly variable across vertebrates (Bergmann *et al*., 1984; Grier, 1993, França *et al*., 2012). Tight junctions and desmosomes are usually observed between adjacent Sertoli cells in anamniotes (fish and amphibians), and the barrier is present after meiosis is complete and the cyst is composed by early spermatids, the haploid cells (Pudney, 1993; McClusky, 2006; Batlouni *et al*., 2009; Schulz *et al*., 2010; França *et al*. 2012). However, a study in zebrafish showed that lanthanum, a tracer widely used to investigate barrier effectiveness, is never observed in the seminiferous tubule lumen, even when there is no functional barrier present (Leal *et al*., 2009). Similar to mammals, in reptiles and birds the barrier seems to be formed immediately after the onset of meiosis; however, there is still little information regarding its composition and structure (Bergmann *et al*., 1984; Bergmann and Schindelmeiser, 1987; Gribbins, 2011; Cheng and Mruk, 2012; Ahmed *et al*., 2016). 

## The transition region

The dogma that the adult Sertoli cells population constitutes a terminally differentiated population in mammals has been challenged by several recent studies (Hayrabedyan *et al*., 2012; Haverfield *et al*., 2015; Figueiredo *et al*., 2016; França *et al*., 2016). Therefore, the possible existence of a progenitor Sertoli cell has been strengthened by new evidences raised from sexually mature laboratory rodent models, indicating that the transition region, where the seminiferous tubules connect to the *rete testis*, could be a specific area for immature Sertoli cells (Tarulli *et al*., 2013; Figueiredo *et al*., 2016; Kulibin and Malolina, 2016). This assumption is supported mainly by current molecular observations showing that a subpopulation of Sertoli cells within the transitional region are mitotically active (BrdU-positive; Cyclin-D1-positive; Ki-67-positive) and that they do not express typical differentiated Sertoli cells markers such as the transcription factor GATA-4 and the androgen receptor (Tarulli *et al*., 2013; Aiyama *et al*., 2015; Figueiredo *et al*., 2016; Kulibin and Malolina, 2016). Moreover, it has already been described that the adult Sertoli cells population is not morphologically homogeneous. In this particular aspect, the transition region presents modified Sertoli cells that exhibit features that resemble undifferentiated Sertoli cells, such as the presence of more ovoid nucleus, with less indentations, smaller nucleolus and more peripheral heterochromatin (Dym, 1974; Osman, 1978; Nykänen, 1979). More recently, it has been shown that these modified Sertoli cells divide in culture and are able to form colonies and generate cord-like structures (Kulibin and Malolina, 2016). Because this particular area of mammalian testis also contains spermatogonial stem cells, it has been suggested that the transition region might be an area where the seminiferous tubules continues to grow in sexually mature individuals (Tarulli *et al*., 2013; Aiyama *et al*., 2015; Figueiredo *et al*., 2016). Moreover, this assumption is corroborated by new findings indicating that the transition region is a site where seminiferous tubules are originally formed (Malolina and Kulibin, 2017).

Similar to horses and showing a testis gradient during postnatal testis development, in pigs the testis parenchyma grows asynchronously, starting its maturation nearby the intermediate and the central (transitional) area, where the testis mediastinum and the *rete testis* are located (Avelar *et al*., 2010). Moreover, resembling sexually mature laboratory rodents, Sertoli cell mitotic activity in pubertal pigs was higher in the transitional region, which is probably a primary site of seminiferous tubules growth in length. In some teleost orders, there are also evidences of the existence of functionally different testicular regions. For instance, once this specific area presents restricted distribution of proliferative Sertoli cells associated with immature spermatogonia, in perciform fish it was demonstrated that the distal region of the seminiferous tubules, nearby the tunica albuginea (blind end region), might be the source of new spermatogenic cysts formation (Schulz *et al*., 2005). Considering that the above-mentioned region present immature Sertoli cells exhibiting high proliferative potential, one could hypothesize the existence of stem Sertoli cells, an important aspects that still needs further investigation.

It is interesting to mention that in some invertebrate species, such as flies and nematodes, the germline stem cell niche is located at the terminal end of the tubular aspect of the gonads. Therefore, in this location, undifferentiated spermatogonial cells are steadily maintained (de Cuevas and Matunis, 2011; Aiyama *et al*., 2015). Another important aspect regarding the germline stem cell niche in these invertebrates is the presence of proliferative somatic cyst progenitor cells (similar to the transition region in mammals?), which produce distally somatic cyst cells that support the development and differentiation of germ cells (Dansereau and Lasko, 2008). Therefore, there are some evidences indicating the existence of a stem Sertoli cell pool, which could be a well-conserved feature during evolution. 

## Relationship between Sertoli and germ cells

### Cystic vs Non-Cystic spermatogenesis

The maintenance of continuous sperm production throughout life in males is very complex and the fine regulation (protein synthesis and signaling) of spermatogenesis is under constant investigation (Wong and Cheng, 2009; Ramaiah and Wilkinson, 2015; Yang and Oatley, 2015; França *et al*., 2016; Griswold, 2016). In this context, although showing particularities among different vertebrate groups, the interactions between Sertoli and germ cells is crucial for the development and completion of spermatogenesis (Hess and França, 2007). In amniotes (reptiles, birds and mammals), a single and non-dividing Sertoli cell supports the development of different germ cells at the same time (non-cystic pattern of spermatogenesis) (Fig. 1; Russell and Griswold, 1993; França *et al*., 2015). In particular, at their different areas/regions the Sertoli cells present the following contacts/functions: i) regulate spermatogonia self-renew or differentiation in the basal compartment of the seminiferous epithelium (de Rooij, 2001); ii) create contact with spermatocytes on its lateral side, regulating the meiotic process from the duplication of DNA content to the formation of spermatids (Russell, 1977); iii) interact specifically with spermatids in the adluminal/apical portions, regulating their morphology, the reabsorption of residual bodies and controlling spermiation (Meistrich and Hess, 2013). 

Different from the seminiferous epithelium organization cited above, in fish and amphibians (anamniotes) the cystic type of spermatogenesis is observed (Figure 1; França *et al*., 2015). In these vertebrate groups, spermatogenic cysts are formed when Sertoli cells surround the type A spermatogonia. From this point, Sertoli cells synchronously coordinate the development of these cells until they differentiate into sperm (Schulz *et al*., 2010, França *et al*., 2015). Thus, in this arrangement, a histological testis section is composed of different spermatogenic cysts, i.e., distinct units of germ cells in the same stage of development enveloped by groups of Sertoli cells (Leal *et al*., 2009). It is believed that in this cystic organization the Sertoli cells are more efficient, meaning that more germ cells can be supported by Sertoli cells during their development, allowing a higher sperm production (França *et al*., 2015). Moreover, it is hypothesized that this cystic structure allows the concentration of specific factors required for each phase of spermatogenesis (spermatogonial, spermatocitary, and spermiogenic), resulting in lower germ cell apoptosis (Vilela *et al*., 2003; França *et al*., 2015). 

As it was already mentioned, due to continuous testis growth during adulthood, another characteristic already described in cystic spermatogenesis is the capacity of Sertoli cells to divide in adult animals, where Sertoli cells are observed proliferating mainly when they are in contact with mitotically active spermatogonia and in association with spermatocytes (França *et al*., 2015). Although there is still no proof regarding the existence of Sertoli stem cell in fish testis, some authors believe in the existence of a stem cell population, giving rise to somatic Sertoli cells (Morais *et al*., 2013; França *et al*., 2015).

### Spermatogonial stem cell’s niche

The Sertoli cells play a key role in the functional regulation of spermatogonial stem cells niche, where other somatic testicular cells (Leydig, peritubular myoid cells and macrophages), extracellular matrices and soluble factors actively participate in the complex interaction/signaling with these stem germ cells (Chiarini-Garcia *et al*., 2001; Oatley and Brinster, 2012). In this microenvironment, depending on the stimulus, a balance between self-renewal and differentiation factors regulates the fate of these cells that are capable of self-renewal, differentiation and/or entering into apoptosis (Oatley *et al*., 2011). Acting through Sertoli cells, the glial cell-line derived neutrophic factor (GDNF) and fibroblast growth factor 2 (FGF2) are among the several factors considered important for the regulation of spermatogonial stem cells niche (Tadokoro *et al*., 2002; El Ramy *et al*., 2005; Simon *et al*., 2007; Chen and Liu, 2015; Chen *et al*., 2016; Potter and DeFalco, 2017). In this particular aspect, the total number of Sertoli cells per testis determine the number of available spermatogonial stem cell niches and, consequently, these somatic cells dictates the magnitude of sperm production capacity (Oatley *et al*., 2011). 

Nowadays, two types of spermatogonial stem cell niche are being proposed. In the first type, called closed niche, these undifferentiated cells are concentrated in a particular testicular parenchyma region; whereas in the second type, called opened niche, spermatogonia present a specific distribution inside the seminiferous tubules (Aiyama *et al*., 2015; Yoshida, 2016). In mammals, recent data have demonstrated that the transition region between seminiferous tubules and the *rete testis* is the closed niche area. The Sertoli cells in this region produce high amount of GDNF, maintaining the neighboring spermatogonia in an undifferentiated state (Aiyama *et al*., 2015). Using different species models, several studies have demonstrated that spermatogonial stem cells are usually located in the seminiferous tubules area facing blood vessels of the testis interstitial compartment. It is speculated that FSH, coming from the blood vessels, stimulates the GDNF synthesis of surrounding Sertoli cells (de Rooij, 2009). 

In fish, the distribution of spermatogonia along the testis parenchyma present a very high variation. For example, similar to mammals, in tilapias the closed and opened niche are normally observed and a restricted spermatogonial distribution (closed niche) is characterized by the presence of spermatogonial stem cells that are located in the distal blind end area of the seminiferous tubules nearby the tunica albuginea (Vilela *et al*., 2003; Lacerda *et al*., 2014). However, further studies are still necessary to investigate whether the secretion of GDNF is increased in this region. Also, as it occurs in mammals, in tilapias an unrestricted distribution of undifferentiated/stem spermatogonial cells is observed and these cells are frequently observed in regions of the seminiferous tubules that are facing the blood vessels located in the intertubular compartment **(**Lacerda *et al*., 2014**).**

Because it allows a broad view of testis function, comparative reproductive biology studies are a powerful tool. In these comparative investigations, it was found that typical proteins that are expressed by undifferentiated spermatogonia in mammals, such as OCT4, NANOS2, PLZF and GFRA1, are also expressed in some fish species already investigated (Lacerda *et al*., 2014). The GFRA1 is a membrane receptor involved in spermatogonial self-renewal and its ligand (GDNF), the most extensively studied niche factor, is produced by Sertoli cells. Recent studies have demonstrated that GDNF can promote high proliferation of spermatogonial stem cells *in vitro* in mammals and fish (Aponte *et al*., 2008; Gautier *et al*., 2014). The secretion of GDNF by Sertoli cells is cyclic and, in mammals, coincident with the differentiation of spermatogonial stem cells to type A differentiated spermatogonia that are committed to spermatogenesis, the lowest values of this peptide are found in stages near spermiation (Johnston *et al*., 2011). Therefore, this Sertoli cell regulation ensures a proper gem cell homeostasis and regulates the germ cell density observed in the seminiferous epithelium. Other important factors produced by Sertoli cells are leukemia inhibitory factor (LIF) and WNT5A, essential peptides that promote spermatogonial stem cell survival (Oatley and Brinster, 2012; França *et al*., 2016).

The possible role of different cells in regulating spermatogonial stem cell niche can be observed investigating different animal models, with peculiar testis parenchyma cytoarchitecture. For instance, because differentiated spermatogonia was found preferentially facing Leydig cells cords, studies in the collared peccary allowed to demonstrate that products from Leydig cells, probably androgens, act as a spermatogonial stem cells pro-differentiation factor (Campos-Junior *et al*., 2012). In the scorpion mud turtle testis, spermatogonial stem cells were located close to lymphatic vessels and blood vessels (Costa *et al*., 2018). In horses, these stem cells were located far from the connective tissue (Costa *et al*., 2012), whereas in chinchilla more spermatogonial stem cells are produced after the establishment of puberty, leading to a gradual and striking increase in Sertoli cell efficiency and sperm production after puberty (Leal and França, 2009).

### Spermatogenic efficiency

The relative mass of tubular compartment in the testis determines the space devoted to sperm production (Hess and França, 2007). Thus, in general, species with high proportion of seminiferous tubules present high sperm production and, besides the influence of Sertoli and germ cell factors, the number of Sertoli cells per testis is considered one of the most important determinant of the magnitude of sperm production (Cooke *et al*., 2005; Hess and França, 2007; Lara *et al*., 2018b). In another important aspect, Sertoli cells show distinct capacities to support germ cell development and each Sertoli cell is able to support a relatively fixed, species-specific, number of germ cells. For instance, whereas chinchilla Sertoli cell can support 14 spermatids, each human Sertoli cell is able to support only 3 spermatids, resulting respectively in a huge difference in daily sperm production per testis gram (~60 vs 4-4.5 million) between these species (Hess and França, 2007; Lara *et al*., 2018b). The size of the Sertoli cells and, as a consequence, the space that they occupy in the seminiferous epithelium is another important factor to be considered. Species with reduced Sertoli cells occupancy in the seminiferous epithelium, such as mice (~15%), present higher Sertoli cell and spermatogenic efficiencies when compared to humans, whose Sertoli cells show high occupancy (~40%) in the seminiferous epithelium (Hess and França, 2007).

In relation to the germ cells, the number of differentiated spermatogonial generations, which is phylogenetically determined, is also crucial in determining the magnitude of sperm production. For example, in vertebrates the number of spermatogonial generations varies from around ten in fish to two in humans (França *et al*., 2016). Additionally, particularly in mammals, germ cell loss, which is quite frequent during the spermatogonial (density-dependent regulation) and meiotic (DNA damage) phases of spermatogenesis, also significantly influences the total sperm output (Russell *et al*., 2002; Shaha *et al*., 2010; Aitken *et al*., 2011; Murphy and Richburg, 2014). The spermatogenic cycle length, which is controlled by the germ cell genotype (França *et al*., 1998), is another key factor in determining the efficiency of spermatogenesis (Hess and França, 2007). Considering the majority of the mammalian species already investigated, each spermatogenic cycle lasts about 9 to 12 days, whereas the total duration of spermatogenesis (that takes approximately ~4.5 cycles) lasts approximately 40 to 54 days. The faster the cell differentiation occurs from spermatogonia to spermatozoa, the higher the daily sperm production is. Particularly in humans, another factor that contributes to the lower sperm production is the quite long duration (~70 days) of spermatogenesis (Hess and França, 2007). In fish, the duration of spermatogenesis is very short and is influenced by the water temperature (Egami and Hyodo-Taguchi, 1967; Billard, 1990; Shimizu, 2003; Vilela *et al*., 2003; Nóbrega *et al*., 2009), meaning that higher temperature accelerates germ cells pace during spermatogenesis (Vilela *et al*., 2003; Lacerda *et al*., 2006; Alvarenga and França, 2009; Nóbrega *et al*., 2009).

During evolution, considering the different vertebrates groups, spermatogenic efficiency continually reduces and this characteristic is highly associated with the Sertoli cell support capacity, which decreases from around 100-150 (in fish) to 3 (in humans) spermatids for each Sertoli cell (França *et al*., 2015). In general, the support capacity of this cell in anamniotes is at least 10 times higher than that observed in mammals (França *et al*., 2015). Once more, these findings reinforce that Sertoli cell efficiency is critically important in determining the magnitude of sperm production (Hess and França, 2007), and claim our attention to the fact that perhaps in a near future humans will not produce sperm anymore. 

### Sertoli-Germ cell junctions

Interactions among testicular cells, in particular between Sertoli and germ cells, are crucial to maintain and regulate spermatogenesis in a very coordinated and organized manner, providing all the necessary structural and nutritional support for the developing germ cells (França *et al*., 2016; Cheng and Mruk, 2015; Lara *et al*., 2018a). Therefore, on its basal side Sertoli cells contact spermatogonia through adherens junctions, guiding their homing, niche and colonization (Lara *et al*., 2018a). In Chinese soft-shelled turtle, extensive adherens junctions were also observed between Sertoli and germ cells during active spermatogenesis, preventing the sloughing of germ cells from the epithelium (Ahmed *et al*., 2016). Desmosomes and gap junctions are also observed contacting adjacent germ cells, such as spermatocytes and early spermatids. At their adluminal aspect, Sertoli cells contact elongated spermatids through ectoplasmatic specialization, organizing the movement of these haploid cells as well as their release during spermiation (Cheng and Mruk, 2012; Lara *et al*., 2018a). Different from mammals, ectoplasmic specializations in fish seem to occur only in species with elongated spermatozoa, and this observation lead us to believe that this specialization has a role in the process of spermatid elongation (Batlouni *et al*., 2009). Probably being involved in the maturation of spermatids and similar to mammals, tubulobulbar complexes were described in repiles (Ahmed *et al*., 2016). 

Present between Sertoli cell and germ cells, intercellular channels composed of gap junctions are essential to maintain the metabolic coupling and cell signaling. One of the most studied constitutive protein of the gap junction is connexin 43 (Kidder and Cyr, 2016). In humans (Defamie *et al*. 2003) and rats (Batias *et al*. 2000), connexin 43 is observed in Sertoli, spermatogonia and spermatocytes cells, which suggests an accurate communication among these cells. Similarly, in catfish (*Pseudoplatystoma fasciatum*) connexin 43 was observed in Sertoli cells and inside the germinal cysts containing spermatogonia and primary spermatocytes (Batlouni *et al*. 2005). In both fish and mammals, the expression of connexin 43 in Sertoli cells varies according to the stage of germ cell development, suggesting that a particular group of germ cells can modulate this protein expression in somatic cells (Pointis *et al*., 2010). However, in rainbow trout testis connexin 43 is temporally expressed in some specific spermatogenic cysts (de Montgolfier *et al*., 2007), whereas in guinea pig and mink this protein seems to play a role during the translocation of early spermatocytes into the adluminal compartment (Pelletier, 1995).

## Concluding remarks

Although most available studies are focused on few mammalian species, particularly laboratory rodents, in this review we attempted to highlight and discuss several key aspects related to Sertoli cell biology in vertebrates. From the several topics and parameters evaluated, we can observe that, in order to accomplish their key functions in supporting the development of full spermatogenesis across vertebrates, Sertoli cells present huge and intrinsic plasticity, especially when cystic and non-cystic spermatogenesis is compared. In particular, besides the importance of knowing the regulation of Sertoli cells functions, the better understanding of the fine mechanisms related to Sertoli cell proliferation and efficiency, as well as for spermatogonial stem cell niche regulation, would significantly improve our knowledge that could be applied for instance to conservation and biotechnological approaches. Importantly, we would also greatly benefit from investigations using different experimental models, in which we could address for instance the intricate relationship between Sertoli and germ cells, mainly now that there is a big concern regarding the decline in human sperm counts caused by a multitude of factors.
